# Acrocyanosis Secondary to Esophageal Cancer

**DOI:** 10.1007/s11606-022-07854-1

**Published:** 2022-11-04

**Authors:** Yuichiro Haba, Toshio Naito

**Affiliations:** Department of General Medicine, Juntendo University School of Medicine, Tokyo, Japan

**Fig. 1:**
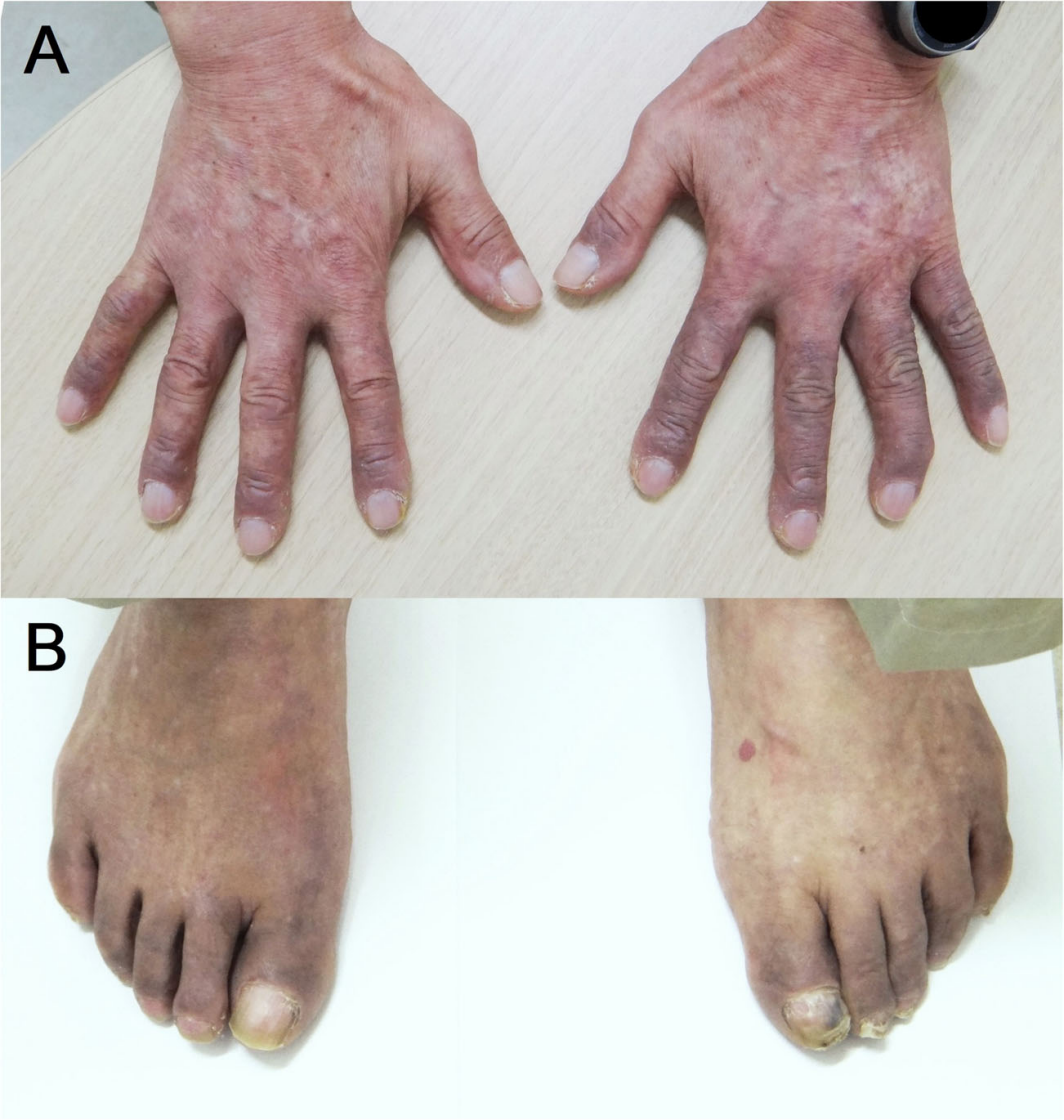
Purple discolorations on initial visit. **A** Fingers; **B** toes

A 76-year-old man presented with a 1-day history of discoloration of fingers and a 1-week history of cough and sore throat. Physical examination revealed purple discolorations on fingers (Fig. 1A) and toes (Fig. 1B). Computed tomography of the chest, looking for causes of the symptoms, showed esophageal wall thickening and possible findings of metastatic cancer. Upper endoscopy revealed esophageal squamous cell carcinoma. The patient’s discolored fingers were diagnosed as acrocyanosis based on its appearance and the history.

Acrocyanosis is characterized by persistent cyanosis mainly in the peripheral extremities. It is generally painless but may be accompanied by moist skin and aggravated by cold exposure.^1, 2^ It is difficult to distinguish primary (no known cause) acrocyanosis to secondary by appearance alone. Secondary acrocyanosis relates to many conditions including malignancies, hemopathy, neurologic disorders, psychiatric disorders, and medications.^1, 2^ He was diagnosed with secondary acrocyanosis due to esophageal cancer. Acrocyanosis secondary to malignancy can also be classified as a paraneoplastic vascular syndrome which may manifest with acrocyanosis, gangrene, and/or Raynaud’s phenomenon.^3^ Clinicians should consider conducting a survey for secondary causes if acrocyanosis is observed.
